# Hyperbaric Oxygen Therapy in Ischaemic Foot Ulcers in Type 2 Diabetes: A Clinical Trial

**DOI:** 10.2174/1874192401812010080

**Published:** 2018-08-31

**Authors:** Sarah Perren, Alfred Gatt, Nikolaos Papanas, Cynthia Formosa

**Affiliations:** 1 Faculty of Health Sciences, University of Malta, Msida, Malta; 2 Diabetes Centre-Diabetic Foot Clinic, Second Department of Internal Medicine, Democritus University of Thrace, Alexandroupolis, Greece

**Keywords:** Hyperbaric oxygen therapy, Diabetic foot ulcer, Ischaemia, Peripheral arterial disease, Standard wound care, HBOT

## Abstract

**Background and Aims:**

Several treatment modalities and protocols for ischaemic foot ulcers are available. However, little consensus exists on optimal treatment. The aim of this study was to compare Standard Wound Care (SWC) alone* vs. *SWC with adjunct hyperbaric oxygen therapy (HBOT) in the treatment of ischaemic Diabetic Foot Ulcers (DFUs).

**Patients and Methods:**

Twenty-six patients with Type 2 Diabetes Mellitus (T2DM) presenting with a newly diagnosed ischaemic foot ulcer were included. These were divided into group A (SWC with adjunct HBOT) and group B (SWC only). Participants were followed every week for 4 weeks and their ulcers were measured for their surface area and depth to assess any change in wound size.

**Results:**

Both treatment arms succeeded in reducing ulcer area and depth (p<0.001). However, ulcer area (p<0.001) and depth (p<0.001) exhibited superior improvement in group A.

**Conclusion:**

Adjunctive HBOT appears to improve wound healing in ischaemic DFUs and merits further study.

## 1 INTRODUCTION

Lower-extremity complications in patients with diabetes mellitus (DM) have become an increasingly significant public health concern [1]. The global diabetic foot ulcer (DFU) prevalence was 6.3% (95% confidence interval (CI): 5.4-7.3%), which was higher in males (4.5%, 95%CI: 3.7-5.2%) than in females (3.5%, 95%CI: 2.8-4.2%), and higher in patients with type 2 (6.4%, 95%CI: 4.6-8.1%) than those with type 1 DM (5.5%, 95%CI: 3.2-7.7%) [2]. DFUs precede 84% of lower extremity amputations, are associated with increased mortality (by 2.4%) [3-5] and have a high recurrence rate [6], with essentially a poor prognosis [7].

Different local wound management options exist, ranging from a number of dressings to a vast range of advanced treatments used in adjunct to standard treatment of wound care [8]. However, to date, there is no unequivocal evidence for the best local treatment of DFUs [9, 10].

Current Standard Wound Care (SWC) protocols include wound cleansing, optimised glycaemic control, treating infection, improving blood supply and relieving pressure on the wound site [10, 11]. Ideally, all natural phases of wound healing, *i.e.* inflammatory, proliferative phase and maturation phase, which are all oxygen-dependent, should be supported, but this fails to be the case in clinical reality [12]. In an endeavour to improve healing rates, Hyperbaric Oxygen Therapy (HBOT) has been used [13]. However, its use remains debated, and the evidence for its effectiveness, at least in non-ischaemic diabetic ulcers, is limited [14, 15]. Therefore, the aim of this study was to compare the efficacy of HBOT added to SWC* vs. *SWC alone in promoting healing of ischaemic DFUs.

## 2 PATIENTS AND METHODS

This single-centre clinical trial included 26 subjects with Type 2 DM (T2DM) presenting to a tissue viability unit with a newly diagnosed ischaemic DFU. The study was approved by the institutional Ethics Committee and carried out in accordance with the Declaration of Helsinki, as revised in 2000. All patients gave their informed consent. Inclusion criteria were: adult T2DM and newly diagnosed ischaemic DFU. Exclusion criteria were: neuropathic DFU, infected DFU, severe ischaemia requiring urgent revascularisation, recent change of medication, chronic alcohol abuse, systemic disease or pernicious anaemia.

Patients were randomised into group A (n=13; SWC with adjunct HBOT) and group B (n=13; SWC only). Patients were matched for age, gender, DM duration, current medication, HbA_1c_, wound size and location (according to the angiosomes supplying the wound area with arterial blood) [16], using frequency distribution matching to ensure both groups were comparable at baseline. All examinations were carried out by the same investigator to ensure uniformity. Room temperature was kept at 21-23°C to avoid vasoconstriction of digital arteries. Screening process involved review of the patient’s medical history and a lower-extremity physical examination.

### 2.1 Peripheral Sensory Neuropathy

Semmes-Weinstein 10 g monofilaments were used to detect peripheral sensory neuropathy. Testing was performed on the plantar aspect of the hallux and third digit, as well as on the 1^st^, 3^rd^ and 5^th^ metatarsal heads.

### 2.2 Peripheral Arterial Disease

Vascular assessment was carried out by Spectral Waveform Doppler Analysis (SWDA) and Ankle Brachial Pressure Index (ABI). Peripheral arterial disease (PAD) was diagnosed by measurement of ankle brachial index (ABI) and quantitative pedal waveform analysis was obtained. A handheld continuous wave Doppler with an 8MHz probe (Huntleigh, Cardiff, UK) was used. Waveforms were classified as triphasic, biphasic, monophasic discontinuous, and monophasic continuous. Triphasic waveforms were considered as normal, whereas biphasic, monophasic discontinuous, and monophasic continuous waveforms were interpreted as abnormal [16]. Measurements were carried out after patients rested for 5 min in the supine position. ABI 0.9-1.29 was normal [16]. PAD was defined an ABI ≤ 0.89 in either foot. ABI ≥ 1.3 was considered indicative of vascular calcification [17].

DFUs were categorised according to their type, and only ischaemic ones were included. To ensure this, patients were recruited if they exhibited biphasic/monophasic waveforms and/or ABI was <0.9 [17, 18].

### 2.3 Procedure for Wound Measurement

Acetate tracing paper with a printed grid was used to calculate wound area. Sterile probes were used to calculate wound depth. A fine-tipped permanent marker was used to trace the wound outline. Minimal pressure was applied while tracing to prevent distorting the shape and border of the ulceration. A 0.2 cm2 grid was used to calculate the area of the ulcer to increase precision. The Wagner grading system was used to grade ulcerations [19]. In this study, ulcerations included were recorded as a grade 1, 2 or 3.

### 2.4 Treatment Procedure

In group A, 100% oxygen under increased atmospheric pressure was delivered to the lungs of participants through a mask in a multi-place chamber 5 days/week (from Monday to Friday) for approximately 2 h daily, for 40 sessions, as per standard practice [13, 14] and according to our hospital HBOT protocols, under qualified medical supervision. Dressings (calcium alginate) were changed 3 times/week at the HBOT unit.

In both groups, SWC was offered at the tissue viability unit. During each visit, the podiatrist cleansed the wound with sterile saline solution, and any superficial dead tissue or callus present at the lesion or in the surrounding areas was debrided with a sterile scalpel. After wound cleansing, calcium alginate was applied. In all patients, wound measurement was conducted after wound cleansing. Dressings were changed every 2 days in both groups.

### 2.5 Statistical Analysis

Analysis was carried out with Statistical Package for Social Sciences version 25 (SPSS; SPSS Inc, Chicago, IL). Data were normally distributed, as shown by the Shapiro Wilks test. The independent sample t-test was employed to compare differences between the 2 groups. Significance was defined at the 5% level (two-tailed p<0.05).

## 3 RESULTS

Patient characteristics are summarised in Table **1**. All patients exhibited monophasic Doppler waveforms denoting severe arterial disease. Table **2** provides details on wound reductions for all participants.

Both groups demonstrated a significant improvement by the end of the trial; Tables **3**-**5**. However, ulcer area (p<0.001) and depth (p<0.001) exhibited superior improvement in group A.

In group A, mean ulcer area (p=0.013) and depth (p<0.001) were significantly smaller than in group B at week 4. There was also a significant difference in mean ulcer depth between the two groups at week 3 (p=0.002). Reduction of wound area (3.75 cm^2^ in group A* vs. *1.05 cm^2^ in group B, p<0.001) and ulcer depth (0.89 cm in group A *vs*. 0.19 cm in group B, p<0.001) were more pronounced in group A.

## 4 DISCUSSION

The present study has demonstrated that adjunct HBOT enhances the reduction of ulcer area and depth at 4 weeks in T2DM patients with ischaemic DFUs. HBOT is known to ensure hyperoxygenation of ischaemic tissue and restoration from hypoxia [13]. Indeed, fibroblasts, endothelial cells, and keratinocytes are replicated at higher rates in an oxygen-rich environment [20]. Moreover, leukocytes kill bacteria more effectively when supplied with oxygen [21]. Furthermore, it has been postulated that HBOT also improves long-term health related quality of life in patients with chronic diabetic foot ulcers possibly attributed to better ulcer healing [22, 23] as has been reported in this study.

New in this study, when compared with previous works, is the elimination of confounding variables known to affect healing by tight control matching of the 2 groups. This represents the main strength of the study, and the authors are confident that the improvement in wound healing was indeed due to the inclusion of HBOT as an adjunct treatment to SWC. Indeed, different wound types, including non-ischaemic ulcers [21] and chronic wound ulcers [24], have been included in prior studies. Conversely, we exclusively evaluated HBOT in newly-diagnosed DFUs.

The limitations of the study include the small number of patients and the absence of sham HBOT. Thus, caution is needed in the interpretation of results.

## CONCLUSION

In conclusion, adjunct HBOT promotes healing of ischaemic DFUs in T2DM patients. Our results add to the growing evidence on the utility of HBOT in the management of DFUs. Nevertheless, optimal patient selection and long-term results with this treatment modality are still needed before its wider use can be advocated.

## Figures and Tables

**Table 1:** Participant characteristics.

		**Standard Wound Care with** **Adjunctive Hyperbaric Oxygen Therapy**		**Standard Wound Care Only**	
**Age**	Less than 70 years	7	26.9%	7	26.9%
	70 years or more	6	23.1%	6	23.1%
**Gender**	Male	10	38.5%	10	38.5%
	Female	3	11.5%	3	11.5%
**Weight**	≤80 kg	5	19.2%	7	26.9%
	>80 kg	8	30.8%	6	23.1%
**Diabetes duration**	<5 years	6	23.1%	6	23.1%
	≥5 years	7	26.9%	7	26.9%
**Diabetes type**	Type 1	0	0%	0	0%
	Type 2	13	50%	13	50%
**Antidiabetic medication**	Metformin	10	38.5%	10	38.5%
	Insulin	3	11.5%	3	11.5%
**HbA_1c_**	<9%	6	23.1%	6	23.1%
	≥9%	7	26.9%	7	26.9%
**Waveforms**	Monophasic	13	50%	13	50%
	Biphasic	0	0%	0	0%
	Triphasic	0	0%	0	0%
**Wagner classification**	Grade 1	2	7.7%	2	7.7%
	Grade 2	2	7.7%	2	7.7%
	Grade 3	9	34.6%	9	34.6%
**Past ulcers**	Yes	11	42.3%	8	30.8%
	No	2	7.7%	5	19.2%
**Smoking history**	Never	7	26.9%	3	11.5%
	Past smoker	5	19.2%	6	23.1%
	Present smoker	1	3.8%	4	15.4%
**Drinking History**	Never	10	38.5%	10	38.5%
	Past drinker	3	11.5%	3	11.56%
	Present drinker	0	0%	0	0%

**Table 2:** Wound reductions.

**Week**		**Range (cm^2^)**	**Mean (cm^2^)**	**Std. Dev.**	**p**
**SWC with adjunctive HBOT**	1	7.79	11.73	2.239	<0.001
	2	7.54	10.14	2.219	
	3	6.64	8.96	1.881	
	4	6.50	7.98	1.951	
**SWC only**	1	3.02	10.60	0.926	<0.001
	2	3.17	10.14	0.891	
	3	2.86	9.78	0.857	
	4	2.80	9.55	0.835	

SWC: standard wound care: HBOT: hyperbaric oxygen therapy.

**Table 3:** Mean depth by weeks and groups.

**Week**		**Range (cm)**	**Mean (cm)**	**Std. Dev.**	**p**
**SWC with adjunctive HBOT**	1	1.50	1.59	0.388	<0.001
	2	1.40	1.31	0.362	
	3	1.00	0.97	0.312	
	4	0.80	0.71	0.253	
**SWC only**	1	1.10	1.53	0.335	<0.001
	2	1.00	1.48	0.322	
	3	1.00	1.40	0.316	
	4	1.00	1.34	0.293	

SWC: standard wound care: HBOT: hyperbaric oxygen therapy.

**Table 4:** Mean surface area by weeks and groups.

	**Group**	**Range (cm^2^)**	**Mean (cm^2^)**	**Std. Dev.**	**p**
Week 1	SWC with adjunctive HBOT	7.79	11.73	2.239	0.112
	SWC only	3.02	10.60	0.926	
Week 2	SWC with adjunctive HBOT	7.54	10.14	2.219	0.996
	SWC only	3.17	10.14	0.891	
Week 3	SWC with adjunctive HBOT	6.64	8.96	1.881	0.173
	SWC only	2.86	9.78	0.857	
Week 4	SWC with adjunctive HBOT	6.50	7.98	1.951	0.013
	SWC only	2.80	9.55	0.835	

SWC: standard wound care: HBOT: hyperbaric oxygen therapy.

**Table 5:** Mean depth clustered by groups and weeks.

	**Group**	**Range (cm)**	**Mean (cm)**	**Std. Dev.**	**p**
**Week 1**	SWC with adjunctive HBOT	1.50	1.59	0.388	0.669
	SWC only	1.10	1.53	0.335	
**Week 2**	SWC with adjunctive HBOT	1.40	1.31	0.362	0.220
	SWC only	1.00	1.48	0.322	
**Week 3**	SWC with adjunctive HBOT	1.00	0.97	0.312	0.002
	SWC only	1.00	1.40	0.316	
**Week 4**	SWC with adjunctive HBOT	0.80	0.71	0.253	<0.001
	SWC only	1.00	1.34	0.293	

SWC: standard wound care: HBOT: hyperbaric oxygen therapy.
